# Does FDG PET-Based Radiomics Have an Added Value for Prediction of Overall Survival in Non-Small Cell Lung Cancer?

**DOI:** 10.3390/jcm13092613

**Published:** 2024-04-29

**Authors:** Andrea Ciarmiello, Elisabetta Giovannini, Francesca Tutino, Nikola Yosifov, Amalia Milano, Luigia Florimonte, Elena Bonatto, Claudia Bareggi, Luca Dellavedova, Angelo Castello, Carlo Aschele, Massimo Castellani, Giampiero Giovacchini

**Affiliations:** 1 Nuclear Medicine Department, Sant’ Andrea Hospital, 19124 La Spezia, Italy; elisabetta.giovannini@asl5.liguria.it (E.G.); francesca.tutino@asl5.liguria.it (F.T.); nikola.yosifov@asl5.liguria.it (N.Y.); giampiero.giovacchini@asl5.liguria.it (G.G.); 2 Oncology Unit, Sant’ Andrea Hospital, 19124 La Spezia, Italy; amalia.milano@asl5.liguria.it (A.M.); carlo.aschele@asl5.liguria.it (C.A.); 3 Nuclear Medicine Department, Fondazione IRCCS Ca’ Granda Ospedale Maggiore Policlinico, 20122 Milan, Italy; luigia.florimonte@policlinico.mi.it (L.F.); angelo.castello@policlinico.mi.it (A.C.); massimo.castellani@policlinico.mi.it (M.C.); 4 Division of Nuclear Medicine, IEO European Institute of Oncology IRCCS, 20122 Milan, Italy; elena.bonatto@unimi.it; 5 Medical Oncology Unit, Fondazione IRCCS Ca’ Granda Ospedale Maggiore Policlinico, 20122 Milan, Italy; claudia.bareggi@policlinico.mi.it; 6 Nuclear Medicine Department, ASST Ovest Milanese, 20025 Legnano, Italy; luca.dellavedova@asst-ovestmi.it

**Keywords:** NSCLC, radiomics, survival, PET, FDG

## Abstract

**Objectives**: Radiomics and machine learning are innovative approaches to improve the clinical management of NSCLC. However, there is less information about the additive value of FDG PET-based radiomics compared with clinical and imaging variables. **Methods**: This retrospective study included 320 NSCLC patients who underwent PET/CT with FDG at initial staging. VOIs were placed on primary tumors only. We included a total of 94 variables, including 87 textural features extracted from PET studies, SUVmax, MTV, TLG, TNM stage, histology, age, and gender. We used the least absolute shrinkage and selection operator (LASSO) regression to select variables with the highest predictive value. Although several radiomics variables are available, the added value of these predictors compared with clinical and imaging variables is still under evaluation. Three hundred and twenty NSCLC patients were included in this retrospective study and underwent 18F-FDG PET/CT at initial staging. In this study, we evaluated 94 variables, including 87 textural features, SUVmax, MTV, TLG, TNM stage, histology, age, and gender. Image-based predictors were extracted from a volume of interest (VOI) positioned on the primary tumor. The least absolute shrinkage and selection operator (LASSO) Cox regression was used to reduce the number of variables and select only those with the highest predictive value. The predictive model implemented with the variables selected using the LASSO analysis was compared with a reference model using only a tumor stage and SUVmax. **Results**: NGTDM coarseness, SUVmax, and TNM stage survived the LASSO analysis and were used for the radiomic model. The AUCs obtained from the reference and radiomic models were 80.82 (95%CI, 69.01–92.63) and 81.02 (95%CI, 69.07–92.97), respectively (*p* = 0.98). The median OS in the reference model was 17.0 months in high-risk patients (95%CI, 11–21) and 113 months in low-risk patients (HR 7.47, *p* < 0.001). In the radiomic model, the median OS was 16.5 months (95%CI, 11–20) and 113 months in high- and low-risk groups, respectively (HR 9.64, *p* < 0.001). **Conclusions**: Our results indicate that a radiomic model composed using the tumor stage, SUVmax, and a selected radiomic feature (NGTDM_Coarseness) predicts survival in NSCLC patients similarly to a reference model composed only by the tumor stage and SUVmax. Replication of these preliminary results is necessary.

## 1. Introduction

Over the past few years, there have been significant advances in the treatment of non-small cell lung cancer (NSCLC). Biological, target, and immunotherapies have been introduced in the clinical routine along with standard treatments, represented by surgery, chemotherapy, and radiotherapy. Despite the wide therapeutic armamentarium available, lung cancer still remains the first cause of cancer-related death worldwide. Moreover, it is evident that patients at the same stage according to the classification of malignant tumors based on tumor size, T, lymph nodes, N, and metastasis, M, (TNM) and with the same treatment may experience different outcomes, suggesting a different biological behavior within the tumor [1].

The ability to predict prognosis on the basis of tumor biology and set up a therapy may be difficult in clinical practice. It is not uncommon that the outcomes of patients with tumors classified in the same TNM staging group may evolve differently. One of the causes is tumor heterogeneity. According to recent research, spatial heterogeneity is correlated with tumor progression, treatment resistance, and recurrence [2]. Radiomics, defined as the process of identifying mineable parameters hidden in the pixels of images and routinely non-detectable by the human eye, is a new technology that potentially could allow to study of tumor heterogeneity and to investigate its role in disease development. Indeed, specific features extracted from images by radiomics analysis (e.g., intensity, shape, and texture) add relevant information, which could be related to other clinical variables [3,4]. Radiomics analysis provides quantitative information based on signal intensity of regions of interest that improves the set of qualitative (semantic) information currently used in clinical practice. Radiomic features extracted from the medical images belong to different categories defined as a function of increasing complexity. The structural ones describe morphological aspects, such as eccentricity, volume, and presence of concavity (solidity), while those of greater complexity are defined according to the organization of spatially contiguous areas with different signal intensities [5]. In recent years, machine learning and radiomic techniques have been increasingly used for whole-body and brain image analysis in oncology and neuroimaging in order to improve diagnostic accuracy for several disorders [6,7]. The enormous amount of data associated with medical images, such as size, morphology, and radiotracer uptake, represents a great source of information where radiomics can play an important role in the detection of quantitative information normally not evaluable by the human eye. In this study, we described a machine learning approach to create a prognostic model for patients with newly diagnosed NSCLC. To explore the additive value of radiomics, we compared the performance of a machine learning radiomic model, integrating the tumor stage, standardized uptake values (SUVmax), and radiomic features, with a reference model integrating only tumor stage and SUVmax for the prediction of overall survival (OS) in NSCLC patients. Our major finding is that the radiomic model did not significantly increase the prediction of survival in NSCLC patients compared with the reference model.

## 2. Materials and Methods

### 2.1. Population

This retrospective study was based on clinical records and F-18 fluorodeoxyglucose positron emission tomography/computed tomography (FDG PET/CT), images of NSCLC patients from two institutions (S. Andrea Hospital, La Spezia, Italy, and IRCCS Ca’ Granda-Ospedale Maggiore Policlinico, Milan, Italy) collected between November 2010 and December 2019.

Patients were retrospectively recruited from (S. Andrea Hospital, La Spezia, Italy, and IRCCS Ca’ Granda-Ospedale Maggiore Policlinico, Milan, Italy). The analysis was performed on clinical records and imaging studies collected between November 2010 and December 2019. The regional review committee granted ethical approval (CER Liguria: 251/2020/10601) for this study (8 June 2020). We de-identified data to avoid any potential breach of patient privacy and processed them for research purposes from 1 July 2020. Inclusion criteria were as follows: (1) histologically confirmed lung adenocarcinoma or squamous cell carcinoma diagnosed on surgical specimen according to the World Health Organization (WHO) classification [8]; (2) whole body FDG PET/CT performed at initial staging; (3) availability of information about survival status. Exclusion criteria were as follows: (i) previous history of other malignant tumors; (ii) anticancer treatment prior to the baseline PET/CT scan; (iii) PET/CT performed not at initial staging. Written informed consent was obtained from all patients before PET/CT imaging procedures. As 1184 out of the 1504 patients initially recruited from the institutional database did not meet inclusion criteria, they were excluded. Therefore, the study cohort consisted of 320 subjects (21% of the initial sample) with histologically confirmed diagnosis of NSCLC, encompassing 219 males and 101 females. Among these, 209 were adenocarcinomas and 111 were squamous cell carcinomas. Patients were classified by a nuclear medicine physician and an oncologist according to the 8th edition of the TNM staging system [9] measuring the tumor size (T) on the unenhanced CT of the PET/CT; the analysis of the presence of regional lymph node involvement (N) and distant metastases (M) was based on visual and semiquantitative evaluation of FDG uptake compared with background without any fixed uptake threshold and cytology/histology, as available.

Overall survival was defined by the time between the date of imaging and that of death or censoring. The date of the last follow-up visit was used for censoring surviving patients. Hospital records were used to assess patients’ status.

All procedures were performed in accordance with institutional and/or national ethical standards and the 1964 Helsinki Declaration and its subsequent amendments.

Informed consent was obtained from all individual participants included in the study.

### 2.2. PET Imaging and Segmentation Procedures

PET/CT imaging was performed on a Discovery 710 (GE Healthcare, Chicago, IL, USA) and a Biograph (Siemens Healthcare, Erlangen, Germany) tomograph according to standard clinical scanning protocol used in the two participating centers. Patients were required to fast for ≥6 h prior to administration of 3.84 MBq/kg body weight of FDG (range, 2.57–4.91 MBq/kg). The mean FDG uptake period was 63 min (range, 47–83 min). Head-to-hip scanning was performed in all patients with the standard protocol using low-dose CT followed by PET acquisition. CT images were obtained at 120 kV and 100 mA, 0.33 s per rotation using a slice thickness of 3.0 mm and reconstructed to a 512 × 512 matrix (voxel size: 0.98 × 0.98 × 3.0 mm^3^). PET imaging was obtained in a three-dimensional mode with 6 to 8 beds and lasting not less than 2 min each for a whole body acquisition from head to hip. PET images were reconstructed with CT-based attenuation correction using an ordered subset expectation maximization (OSEM) iterative algorithm. Final PET images had 256 × 256 matrix size and anisotropic voxels of 2.73 × 2.73 × 3.27 mm^3^. SUVmax was obtained by normalizing the injected activity concentration to the patient’s body weight.

PET images were reviewed in the Nuclear Medicine Unit laboratory of the S. Andrea Hospital by two board-certified nuclear medicine physicians. Fused PET/CT and CT images were processed under PET Volume Computerized Assisted Reporting (PETVCAR version 2.0) commercial software running on Advantage Workstation (version 4.6; GE Healthcare) in order to segment lung cancer. PETVCAR uses an adaptive iterative volume delineation algorithm (AT-AIA), which is able to automatically segment the target volume from background tissue using a SUVmax threshold of 2.5 and a 3D isocontour of 41% of the maximum voxel value measured in the target lesion [10,11]. SUVmax and SUVmean were obtained as the maximum and mean activity of the voxels within the volume of interest. Metabolic tumor volume (MTV) was defined as the total tumor volume contained in the VOI. Total lesion glycolysis (TLG) was calculated as MTV multiplied by the mean SUV of the lesion.

### 2.3. Image Analysis and Textural Features Extraction

Before the extraction, we applied the ComBat harmonization method [12] to remove batch effects from images of different scanners using the “neuroCombat” package in R.

In compliance with protocols described by the Imaging Biomarker Standardization Initiative (IBSI) (https://arxiv.org/abs/1612.07003, accessed on 1 March 2024), intensity discretization and spatial resampling were applied before feature extraction. Images were discretized with a 64 fixed bin width and resampled to 5 mm^3^ voxel size with B-spline interpolation.

Spatial and intensity heterogeneity of FDG PET images were evaluated with two groups of features including (a) intensity and (b) texture. First-order statistics describing the voxel intensities within the VOI mask, including the tumor image, were assessed using SUV metric, including maximum voxel intensity (SUVmax), MTV, and TLG. The target volume was also limited to tumor volume in patients with lymph nodes and distant pathological FDG uptake sites.

Eighty-seven radiomic variables were extracted with the Pyradiomics software package (version 3.1.0) from each lung tumor [13,14].

Among them were 15 first-order, 21 gray-level co-occurrence matrix (GLCM), 16 gray-level run-length matrix (GLRLM), 16 gray-level size zone matrix (GLSZM), 14 gray-level dependence matrix (GLDM), and 5 neighborhood gray-tone difference matrix (NGTDM) textural features. To summarize, the whole set of predictors used for this study included 94 features—namely, 87 radiomic features, SUVmax, MTV, TLG, tumor stage, histology, age, and gender.

### 2.4. Feature Selection and Classification

The survival prediction model was implemented, starting with the whole set of demographic, clinical, radiomic, and molecular variables obtained from PET/CT studies. In order to reduce the number of variables to be included in the model, we used the least absolute shrinkage and selection operator (LASSO) Cox regression analysis [15,16]. The analysis was performed in R 4.1.3 (http://www.r-project.org) with the cv.glmnet package [17].

The model with the best performance was selected using a 10-fold cross-validation and the C-statistic. The detailed description of the method is reported in our previous paper [18].

The model used in subsequent analyses was implemented with the predictors selected from the LASSO Cox regression analysis.

### 2.5. Model Validation and Calibration

Model performance was estimated with the calibration and validation procedures available under the RMS package [19]. The evaluation metric was based on C-index and Brier scores. The calibration method was performed by comparing the predicted to the observed probabilities. Validation was obtained by computing an optimism-corrected C-statistic after 1000 bootstrapped resampling. To test data not included in the training process, we also carried out the validation of the test dataset. The high- and low-risk survival groups were defined using the median individual risk score assessed with the GGrisk package of R software [20]. Kaplan–Meier analysis with the ggsurvplot function and log-rank test was used to evaluate the accuracy of the risk score in identifying patients with a lower probability of survival.

### 2.6. Model Design

Ninety-four predictors were included in Lasso’s initial selection. They included 2 demographic (age and gender), 3 metabolic (SUVmax, MTV, and TLG), 87 radiomic, and 2 clinical features (histology and disease stage). The model was built with all the predictors chosen by Lasso analysis. For comparison, a model including the disease stage only was implemented.

Model performance was assessed on a test dataset obtained by splitting the study sample into training (80%) and test (20%) datasets. Therefore, the performance evaluation was carried out on data excluded from the training.

The ratio used to divide the study population into training and test samples is reported to be useful in developing high-performance predictive models when the sample size is greater than 100 and the percentage of incomplete data is less than 15% [21].

Moreover, the risk prediction model implemented using the disease stage alone was used to assess the added value of the radiomic model to the reference model. Model performance was evaluated by applying estimates derived from the training dataset to the test sample. Supplementary Table S2 reports clinical and demographic characteristics of the study sample by dataset.

### 2.7. Statistics

Statistical analysis and figures were obtained under R software (version 4.1.3, http://www.r-project.org). T-statistic was used for continuous variables. Where appropriate, the degrees of freedom were adjusted for inequality of variance.

Glmnet was used for LASSO regression analysis. Kaplan–Meier was performed under the ggsurvplot R package. The pROC and survival-ROC packages were applied to analyze the area under the receiver operating characteristic curve (AUCs).

Validation plots were produced by the root mean squares (RMS) package. The chi-square analysis was used for categorical variables. Positive predictive value (PPV), negative predictive value (NPV), and their 95% confidence intervals (CIs) were calculated to estimate how strongly the model-predicted diagnosis was associated with clinical outcomes.

The RMS package was used for predictive model validation [22]. The chi-square test was used to measure categorical variables’ association with outcome. Moreover, the predictive model performance was assessed with positive predictive value (PPV) and negative predictive value (NPV) with their 95% confidence intervals (CIs).

In order to evaluate the model’s predictive performance in different disease stages we also split the sample into an early disease group (stages I and II) and an advanced disease group (stages III and IV). The ability of the model to distinguish deceased from survivors was assessed with AUCs. The differences between the AUCs obtained in the two subgroups were evaluated with the DeLong test [23]. Two-sided *p*-values less than 0.05 were considered statistically significant.

## 3. Results

Three hundred and twenty patients were recruited and randomized to include 80% (*n* = 256) in training and 20% (*n* = 64) in test datasets. The distribution of histological types showed no significant differences between the training and test datasets (*p* > 0.05). Patients’ characteristics are summarized in Table 1.

**Figure 1:**
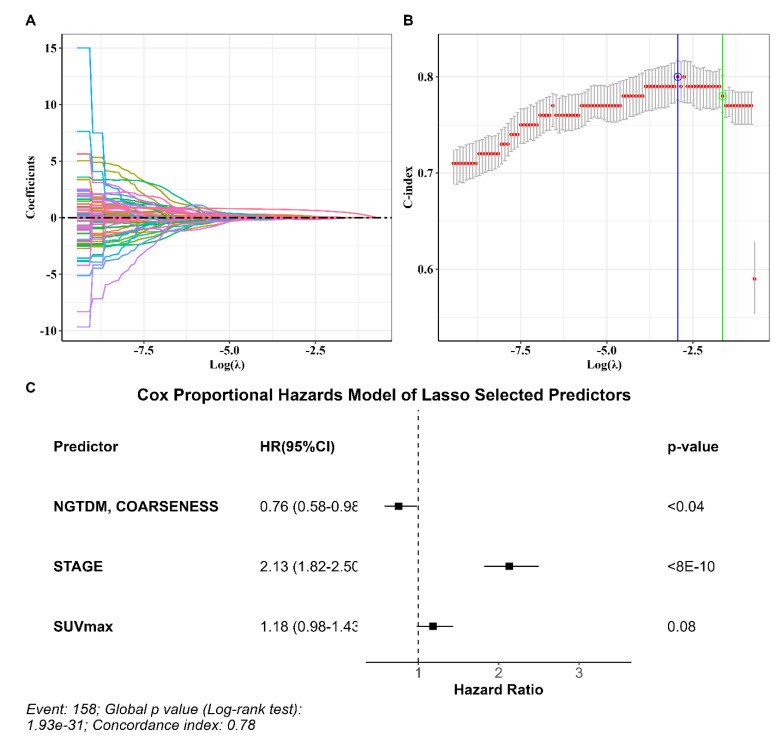
LASSO Cox regression results. (**A**) LASSO coefficients. Coefficient values are plotted against the log (λ). The coefficient of each feature is shown by a colored line. (**B**) C-Index. The plot shows the C-index plotted against log (λ). (**C**) The hazard ratio and 95% confidence interval of predictors survived LASSO selection as estimated by multivariate Cox regression.

The full set of textural, non-textural, and clinical predictors available for each patient consisted of 94 variables. Predictors were selected with 10-fold cross-validation LASSO regression and C-statistic. The parameter producing a C-index within one standard error was 0.195 corresponding to a C-index of 0.782 (standard error = 0.019) (Figure 1). The coefficients of all predictors estimated with the LASSO shrinking algorithm are reported in Supplementary Figure S1.

Three predictors with non-zero coefficients survived the tuning parameters giving the C-index within one standard error of the maximum (Figure 1C). Selected variables included the tumor stage, SUVmax, and the radiomic variable NGTDM_Coarseness (Figure 1C). The LASSO procedure is an automatic operator-independent selection based on a predefined algorithm. Based on the LASSO regression results, two models were created to predict survival in patients with NSCLC: (1) a radiomic model composed using the tumor stage, SUVmax, and NGTDM_Coarseness; (2) a reference model composed using only the tumor stage and SUVmax.

In the training dataset, the concordance between the predicted survival and observed survival curves was detected by the internal validation procedure (Supplementary Figure S2, left panel). The unadjusted and bias-adjusted probabilities were aligned with the line of the best possible correlation (dashed line) between the predicted and observed outcome assessed by the mean absolute error (MAE) of 0.010. Moreover, the scores of 0.791 and 0.159 obtained for the C-statistic and Brier scores, respectively, confirmed the accuracy of the prediction. Validation results on the test set are shown in Supplementary Figure S2, right panel where the scores of 0.015, 0.819, and 0.162 were estimated for the MAE, C-index, and Brier scores, respectively.

**Figure 2:**
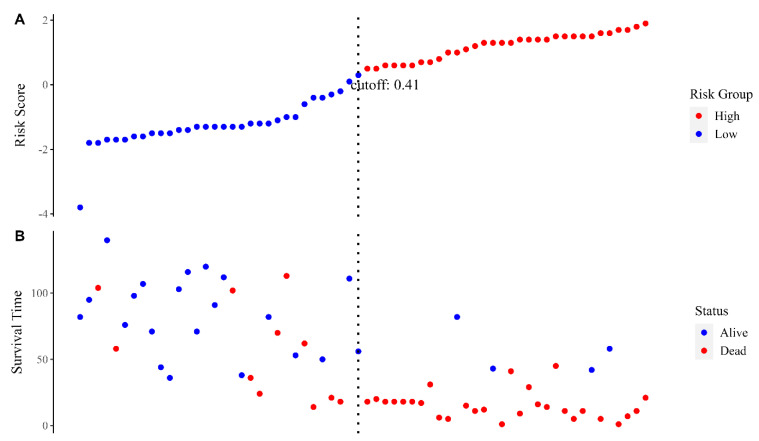
Risk score distribution in the test set. Risk score distribution (**A**), survival status, and survival time (**B**) between the high- and low-risk groups. Using the median risk score (0.41) as a cutoff point, the sample was divided into two groups, defined as high risk and low risk, respectively.

**Figure 3:**
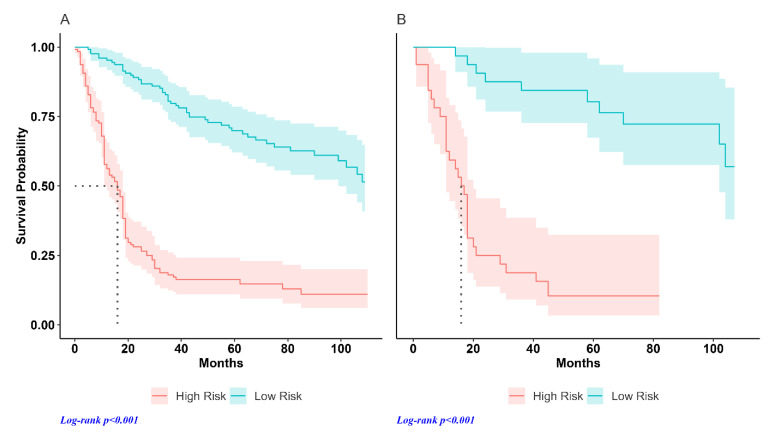
Survival curves for high- and low-risk groups by prediction variable: (**A**) training dataset; (**B**) test dataset.

**Figure 4:**
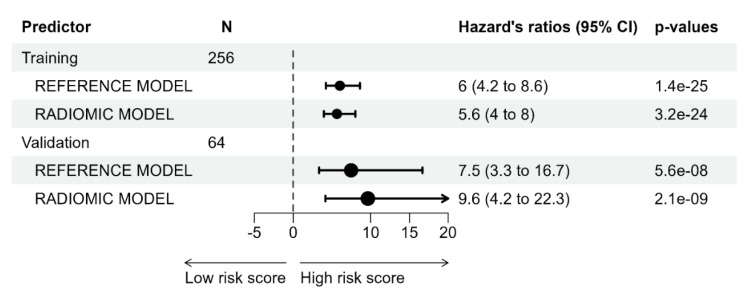
Hazard ratios by model prediction and dataset.

The individual risk score was calculated with Cox regression in both the training and test datasets. Figure 2 shows the distribution of the risk score in the test sample. The results of the Kaplan–Meier analysis performed by dividing the patients of each dataset into high- and low-risk groups are shown in Figure 3. The median OS in the training dataset was 16.0 months in high-risk patients (95%CI, 11–18) and was not reached in the low-risk group (hazard ratio, HR: 5.65, *p* < 0.001) (Figure 3A). In the test dataset, the median OS in the high-risk group was 16.5 months (95%CI, 11–20) and 113 months in the low-risk group (HR 9.64, *p* < 0.001) (Figure 3B). Figure 4 shows the hazard ratio values by dataset and prediction model. The hazard ratio’s *p*-value used to estimate model performance was significant in the training and testing datasets for both prediction models.

The radiomic model correctly classified the majority of deceased and surviving patients into high- and low-risk groups in both datasets. In the training dataset, the radiomic model identified 70% of true positives and 83% of true negatives (OR, 95%CI = 11, 6–20). In the test sample, the model recognized 72% of true positives and 84% of true negatives (OR, 95%CI = 12, 3–40) (Table 2).

**Figure 5:**
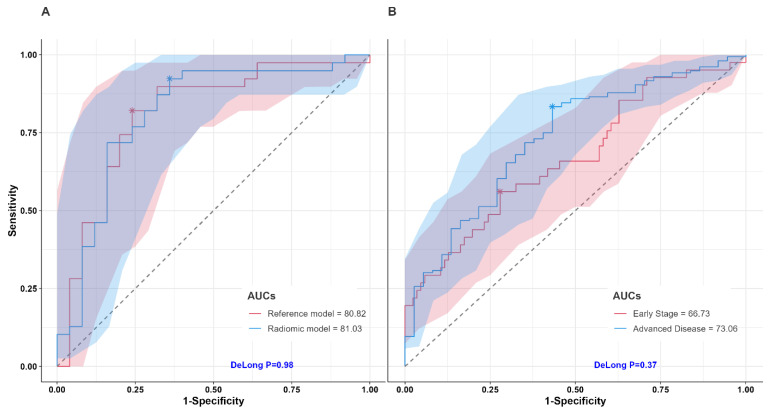
Receiver operating characteristic curves of model performance obtained from the test dataset. (**A**) ROC curves based on reference and radiomic models. (**B**) ROC curves showing the radiomic model performance in patients grouped by disease stage. The asterisk shows the threshold value that maximizes (sensitivity + specificity) for each curve.

The AUCs obtained from the reference and radiomic models were 80.82 (95%CI, 69.01–92.63) and 0.8102 (95%CI, 69.07–92.97), respectively (Figure 5A). The DeLong statistic showed no significant difference between the performance of the two models (*p* = 0.98). Radiomic model performance was also evaluated separately on a subset including only I and II stages (early disease) vs. III and IV stages (advanced disease). The AUCs obtained from the early and advanced disease were 66.73 (95%CI, 56.29–77.16) and 73.06 (95%CI, 64.12–81.99), respectively (Figure 5B). No statistically significant difference between AUCs was shown by the DeLong test (*p* = 0.37).

**Figure 6:**
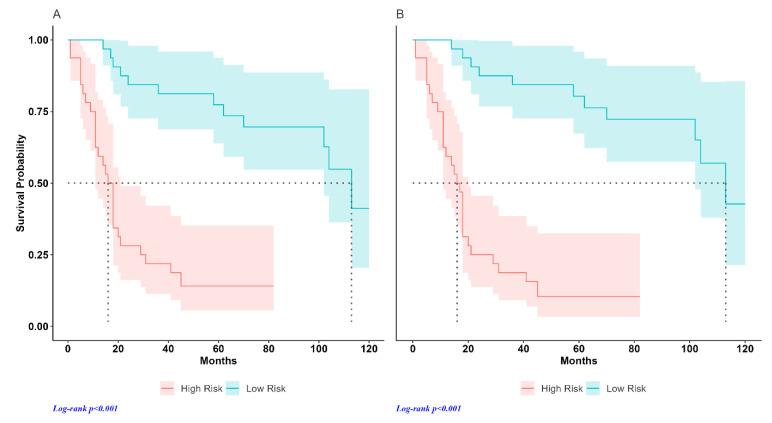
Survival curves for high- and low-risk groups by prediction variable: (**A**) reference model (stage and SUVmax); (**B**) radiomic model (stage, SUVmax and radiomic feature).

Figure 6 shows the results of the Kaplan–Meier analysis obtained using the two models. The median OS in the reference model was 17.0 months in high-risk patients (95%CI, 11–21) and 113 months in the low-risk group (HR 7.47, *p* < 0.001) (Figure 6A). The median OS in the radiomic model was 16.5 months (95%CI, 11–20) and 113 months in high- and low-risk groups, respectively (HR 9.64, *p* < 0.001) (Figure 6B).

The median OS in the advanced disease group was 11 months (95%CI, 10–12) and 21 months (95%CI, 19–42) in high- and low-risk groups, respectively (HR 2.48, *p* < 0.001). The median OS in the early disease group was 113 months in high-risk patients and was not reached in the low-risk group (HR 2.40, *p* = 0.007). Survival estimates in our study are similar to those previously reported in the literature [24,25].

## 4. Discussion

A wide variation in clinical outcomes has been observed in patients within the same tumor stage [26,27]. In order to improve risk assessment, several attempts have been made to develop linear models based on Cox regression [28] and forest plot [29] integrating TNM with the other known prognostic factors in NSCLC, such as age and histology. Nevertheless, these models are characterized by poor performance, probably due to the limited number of features available and to the linear logic that does not take into account the complexity of clinical variables.

In our study, we investigated the potential impact of the prediction of the clinical outcome of a machine learning approach integrating tumor stage and SUVmax with tumor radiomic features extracted by FDG PET from newly diagnosed NSCLC patients. We used Cox regression analysis to predict the risk of mortality using one model integrating one selected PET radiomic feature (NGTDM_Coarseness), SUVmax, and tumor stage, defined as the radiomic model, and one model using only tumor stage and SUVmax, named the reference model. In fact, TNM is the reference standard for NSCLC staging, and SUVmax, which can be very easily and quickly calculated, was consistently shown to predict survival in NSCLC patients [30]. Although radiomics is fascinating and powerful, its application is also cumbersome and time-consuming and it should be preferred or added to established biomarkers for a specific clinical question in case of a significant added value. Unfortunately, in our study, for the prediction of survival in NSCLC patients, the selected radiomic variable did not provide any significant additional value to that provided by the combination of tumor stage and SUVmax.

This study is part of a framework of machine learning emerging applications in clinical oncology. Machine learning has been employed in the context of medical imaging for segmentation and malignancy characterization [31], in histopathology [32], and in the study of biomarkers [33]. Recent studies have begun to explore the potential role of machine learning networks in prognosis, applying machine learning models to predict OS in NSCLC, mainly integrating tumor stage with clinical factors showing promising results [34,35,36,37,38,39,40,41,42,43,44]. However, a possible research gap lies in the fact that although studies indicate the potential of radiomics to predict survival in oncological patients, the clinical settings and the most important radiomic variables for such prediction need to be better established. This motivated our study. The comparison between the reference and the radiomic model differentiates our study from others in the radiomic field. A careful analysis of the papers suggests that the discrepancy between our study and other studies might be related to the different methodologies adopted.

For example, Ahn et al. reported that different radiomic variables derived from pretreatment FDG PET predicted survival in NSCLC patients; however, in their case, only radiomic variables were included in the analysis [35]. Similar univariate approaches were adopted in other positive studies [36,37].

In another group of studies, radiomic features were integrated with other clinical or imaging variables in fully integrated models, but their added value compared with a reference model was not assessed [38,39].

Moran et al. studied radiotherapy, planning CT and PET/CT 39 for stage III NSCLC patients [41]. Seven conventional prognostic factors, including stage (IIIA vs. IIIB), were collected. Patients were divided into two risk groups based on Kaplan–Meier curves. They showed that the discriminatory power was significantly increased when conventional prognostic factors were combined with PET features compared with conventional prognostic factors alone. There was no significant improvement when conventional prognostic factors were combined with CT features. The main difference from our study is that in their study, SUVmax was not included in the analysis—a factor that might contribute to the different results. Contrarily, Oikonomou et al. showed that radiomic features, but not SUVmax, predict OS after radiotherapy in NSCLC, but in their study, the role of tumor stage was not assessed [42].

Results very similar to ours were obtained by Hannequin et al. [40] and Oliveira et al. [43]. Hannequin et al. showed that PET and CT radiomic features have intrinsic power to predict survival, but they do not significantly improve the prediction of OS and progression-free survival in different multivariate models, in comparison to stage and gender [40]. Oliveira et al. found at univariable analysis no significant association between radiomic features predicting either event-free survival or OS [43]. The authors of this multicentric study emphasized the need for standardized protocols. Indeed, it is also possible that the bicentric nature of our study, with some protocol differences, might have increased the variability of the data and partially reduced the statistical power of the radiomic analysis.

To overcome this drawback, the radiomics data were harmonized using the ComBat approach. This method has proven to be useful in harmonizing the radiomic features extracted from PET obtained with different acquisition and/or reconstruction parameters and in facilitating the validation of the radiomic signatures of data obtained from different centers [45].

The study sample was distributed between the two centers (218 and 102 patients). Clinical and demographic characteristics were similar between the two groups (Supplementary Table S1). Survival rates did not differ significantly between the two groups (Log-rank *p* = 0.2).

It might be objected that our results are biased by the fact that the majority of our sample is represented by advanced-stage patients; therefore, the variable stage naturally outweighs the role of radiomics in the determination of survival. However, when the analysis was performed separately for patients with early disease and advanced disease (Figure 5B), the results of the two analyses were similar showing that the accuracy of the model is independent of the disease stage.

The definition of T was based on the measurement of tumor size on the unenhanced CT of the PET/CT. However, stages III and IV are based not only on size but also on the infiltration of various tissues (pleura, vessels), which is difficult to assess on PET/TC. Owing to the retrospective design of the study, contrast-enhanced CT was not available for all patients as it was often performed in other centers. Therefore, the proportion of stages III and IV may have been slightly underestimated.

In this study, we extracted radiomic data only from the primary tumor, excluding lymph nodes and distant metastases, as the radiomic variables can be affected by various factors, which can limit the reproducibility and accuracy of the estimates (lesion size, contouring, and/or harmonization approach [46].

Li et al., using PET/CT, showed that the best classifier for predicting local relapse had only tumor features; in contrast, the best classifier for predicting overall relapse included a node feature [47].

In the study by Carvalho et al., common SUV descriptors were significantly related to OS when extracted from lymph nodes but not from tumor region, while radiomic features were significant when extracted from both lymph nodes and tumor. Therefore, a combination of FDG-PET radiomic features from tumors and lymph nodes was considered desirable to achieve a higher prognostic discriminative power for NSCLC [48]. These preliminary results do not conclude that radiomic analysis is useless, but they show that FDG PET radiomic analysis of only primary tumor provides no additional information in this clinical context. Nowadays, the most common approach in PET studies is to limit radiomic analysis to the tumor. Radiomic analysis of lymph nodes and distant metastases would be more cumbersome but, potentially, also more informative. Similarly, unenhanced, low-dose CT radiomic signature could provide useful information to differentiate and identify histology of lung cancer or to predict survival [18,49,50], but the assessment of CT radiomics was beyond the aim of our study and should be considered as a study limitation.

The main limitation of our study relates to the reproducibility of the results, i.e., test-retest reliability. A sizeable number of factors, such as number of patients, number and type of radiomic variables, software used for data extraction, validation procedure, data harmonization, type of treatment, etc., can all affect radiomic analysis variability. Therefore, replication of our study is warranted, and until such replication is obtained, our results should be considered preliminary. Attention was paid to describing all parts of our methods so that independent replication of our results could be achieved. In future studies, we would like to test the robustness of the current results in a larger population and use a different data selection method.

## 5. Conclusions

We performed a retrospective study, which included 320 NSCLC patients who underwent PET/CT with FDG at initial staging. VOIs were placed on primary tumors only. There is increasing interest in assessing the role of radiomics in predicting survival or other clinical-pathological features in NSCLC [44,51,52,53,54,55,56]. While most studies uniquely tested the role of models mixing clinical and radiomic variables, we performed a comparative study where a model containing uniquely clinical variables (tumor stage and SUVmax), referred to as the reference model, was contrasted with a model that, in addition to tumor stage and SUVmax, contained one radiomic variable (NGTDM_Coarseness, radiomic model); these variables had been automatically selected by our data selection method (LASSO procedure). Our design allowed for assessing the additional value introduced by the radiomic variable. Results showed that the radiomic model predicted survival in NSCLC patients similarly to the reference model. Replication of these preliminary results is warranted to shed more light on the role of radiomic variables in the prediction of prognosis in NSCLC patients.

## Figures and Tables

**Table 1:** Clinical and demographic characteristics of lung cancer patients. ADC = adenocarcinoma, SCC = squamous cell carcinoma, CTx = chemotherapy, RTx = radiotherapy, Sx = surgery.

		Outcome			
Variable	Overall, *n* = 320 ^1^	Alive, *n* = 123 ^1^	Deceased, *n* = 197 ^1^	Statistic	*p*-Value ^2^
**Age**				−2.6	**0.011**
Median (Range)	72 (43, 92)	70 (43, 87)	73 (46, 92)		
**Gender**				4.6	**0.032**
Female	101 (32%)	48 (39%)	53 (27%)		
Male	219 (68%)	75 (61%)	144 (73%)		
**Smoking status**				0.88	**0.35**
Nonsmokers	165 (52%)	68 (55%)	97 (49%)		
Smokers	155 (48%)	55 (45%)	100 (51%)		
**Histology**				20	**<0.001**
ADC	238 (74%)	109 (89%)	129 (65%)		
SCC	82 (26%)	14 (11%)	68 (35%)		
**Stage**				93	**<0.001**
IA, IB	119 (37%)	84 (68%)	35 (18%)		
IIA, IIB	35 (11%)	14 (11%)	21 (11%)		
IIIA, IIIB, IIIC	77 (24%)	17 (14%)	60 (30%)		
IVA, IVB	89 (28%)	8 (6.5%)	81 (41%)		
**Treatment**					**<0.001**
Combined	141 (44%)	41 (33%)	100 (51%)		
CTx	66 (21%)	6 (4.9%)	60 (30%)		
RTx	10 (3.1%)	2 (1.6%)	8 (4.1%)		
Sx	103 (32%)	74 (60%)	29 (15%)		

^1^ *n* (%); ^2^ Welch two-sample *t*-test; Pearson’s chi-squared test; Fisher’s exact test.

**Table 2:** Bivariate analysis of model performance by dataset.

		Observed								
Predicted	*n*	Deceased ^1^	Alive ^1^	χ^2^	*p*-Value ^2^	SS 95%CI ^3^	SP 95%CI ^4^	PPV 95%CI ^5^	NPV 95%CI ^6^	OR 95%CI ^7^
**Training**	256			62	**4.4 × 10^−15^**	70 (62, 77)	82 (73, 89)	86 (79, 91)	62 (54, 71)	10 (5, 18)
High risk		110 (70%)	18 (18%)							
Low risk		48 (30%)	80 (82%)							
**Validation**	64			17	**4.1 × 10^−5^**	72 (55, 85)	84 (64, 95)	88 (71, 96)	66 (47, 81)	12 (3, 40)
High risk		28 (72%)	4 (16%)							
Low risk		11 (28%)	21 (84%)							

^1^ *n* (%); ^2^ Pearson’s chi-squared test; ^3^ sensitivity, confidence interval; ^4^ specificity, confidence interval; ^5^ positive predictive value, confidence interval; ^6^ negative predictive value, confidence interval; ^7^ odds ratio, confidence interval.

## Data Availability

The datasets presented in this article are not readily available because the data are part of an ongoing study.

## Supplementary Materials

The following supporting information can be downloaded at: https://www.mdpi.com/article/10.3390/jcm13092613/s1, Figure S1. LASSO Cox coefficients of all variables used for prediction model implementation; Figure S2. Calibration curves from regression model predictions by datasets; Figure S3. Overfitting impact on prediction model; Table S1. Clinical and demographic characteristics by recruitment center; Table S2. Clinical and demographic characteristics of Lung Cancer patients by dataset.
