# Supplementary material for: Does FDG PET-Based Radiomics Have an Added Value for Prediction of Overall Survival in Non-Small Cell Lung Cancer?
Source: J Clin Med. 2024 Apr 29;13(9):2613. doi: 10.3390/jcm13092613 (PMC11084602; doi:10.3390/jcm13092613)
Supplement: Supplementary file 1 [file jcm-13-02613-s001.zip › Table S1.pdf]

Table S1. Clinical and demographic characteristics by recruitment center.

	Departments			
**Variable**	**DPT#1**, N = 218[1]	**DPT#2**, N = 102[1]	**Statistic**	**p-value**[2]
**Age**	71 (45, 92)	73 (43, 91)	9,930	0.12
**Gender**			0.97	0.3
Female	65 (30%)	36 (35%)		
Male	153 (70%)	66 (65%)		
**Dead**	131 (60%)	66 (65%)	0.63	0.4
**Smoking status**			0.02	0.9
Non smokers	113 (52%)	52 (51%)		
Smokers	105 (48%)	50 (49%)		
**Disease stage**			0.37	0.5
Advanced Disease	129 (59%)	64 (63%)		
Early Stage	89 (41%)	38 (37%)		
**Histology**			1.1	0.3
ADC	166 (76%)	72 (71%)		
SCC	52 (24%)	30 (29%)		

[1] Median (Range); n (%)

[2] Wilcoxon rank sum test; Pearson's Chi-squared test
