# Supplementary material for: Does FDG PET-Based Radiomics Have an Added Value for Prediction of Overall Survival in Non-Small Cell Lung Cancer?
Source: J Clin Med. 2024 Apr 29;13(9):2613. doi: 10.3390/jcm13092613 (PMC11084602; doi:10.3390/jcm13092613)
Supplement: Supplementary file 1 [file jcm-13-02613-s001.zip › Table S2.pdf]

Table S2. Clinical and demographic characteristics of Lung Cancer patients by dataset

		Dataset			
**Variable**	**Overall**, N = 320[1]	**Test**, N = 64[1]	**Train**, N = 256[1]	**Statistic**	**p-value**[2]
**Age**	72 (43, 92)	71 (43, 84)	72 (45, 92)	-1.3	0.19
**Gender**				0.15	0.70
Female	101 (32%)	22 (34%)	79 (31%)		
Male	219 (68%)	42 (66%)	177 (69%)		
**Smoking status**				2.4	0.12
Non smokers	165 (52%)	27 (42%)	138 (54%)		
Smokers	155 (48%)	37 (58%)	118 (46%)		
**Histology**				1.6	0.21
ADC	238 (74%)	52 (81%)	186 (73%)		
SCC	82 (26%)	12 (19%)	70 (27%)		
**Stage**				3.0	0.40
IA, IB	119 (37%)	25 (39%)	94 (37%)		
IIA, IIB	35 (11%)	5 (7.8%)	30 (12%)		
IIIA, IIIB, IIIC	77 (24%)	12 (19%)	65 (25%)		
IVA, IVB	89 (28%)	22 (34%)	67 (26%)		
**Treatment**					0.49
Combined	141 (44%)	23 (36%)	118 (46%)		
CTx	66 (21%)	16 (25%)	50 (20%)		
RTx	10 (3.1%)	2 (3.1%)	8 (3.1%)		
Sx	103 (32%)	23 (36%)	80 (31%)		
**Outcome**				0.01	0.91
Alive	123 (38%)	25 (39%)	98 (38%)		
Deceased	197 (62%)	39 (61%)	158 (62%)		

[1] Median (Range); n (%)

[2] Welch Two Sample t-test; Pearson's Chi-squared test; Fisher's exact test
