# Supplementary figures and images for: Does FDG PET-Based Radiomics Have an Added Value for Prediction of Overall Survival in Non-Small Cell Lung Cancer?
Source: J Clin Med. 2024 Apr 29;13(9):2613. doi: 10.3390/jcm13092613 (PMC11084602; doi:10.3390/jcm13092613)

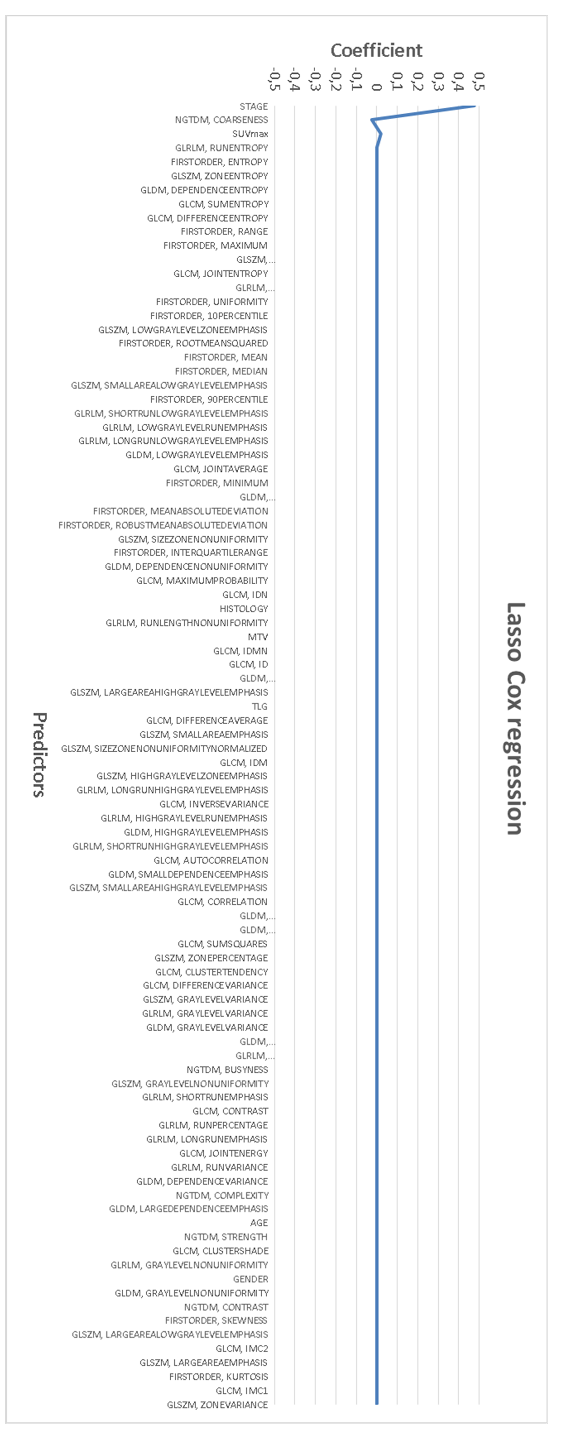

Supplement: Supplementary file 1 [file jcm-13-02613-s001.zip › Figure S1.png]

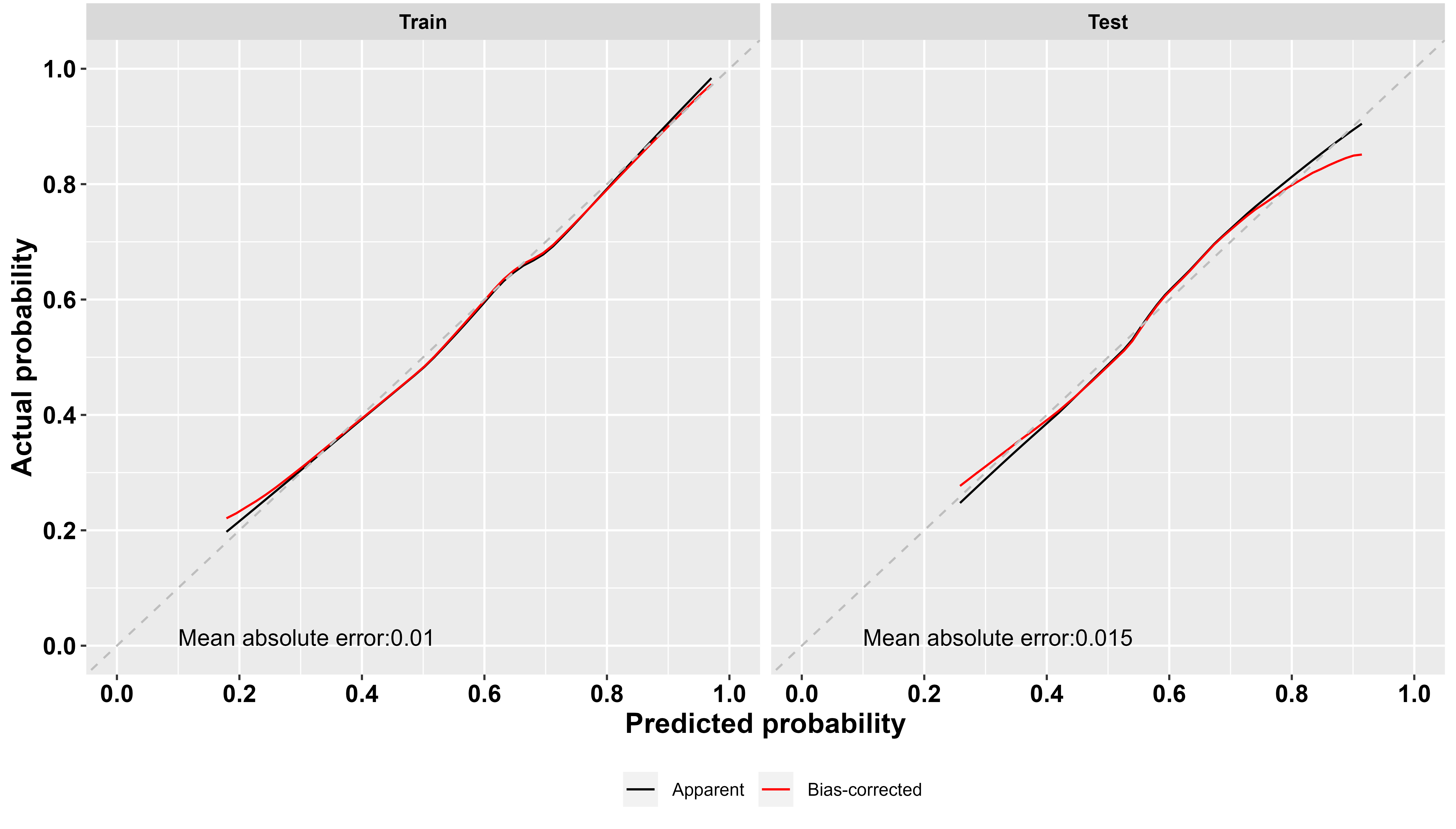

Supplement: Supplementary file 1 [file jcm-13-02613-s001.zip › Figure S2.png]

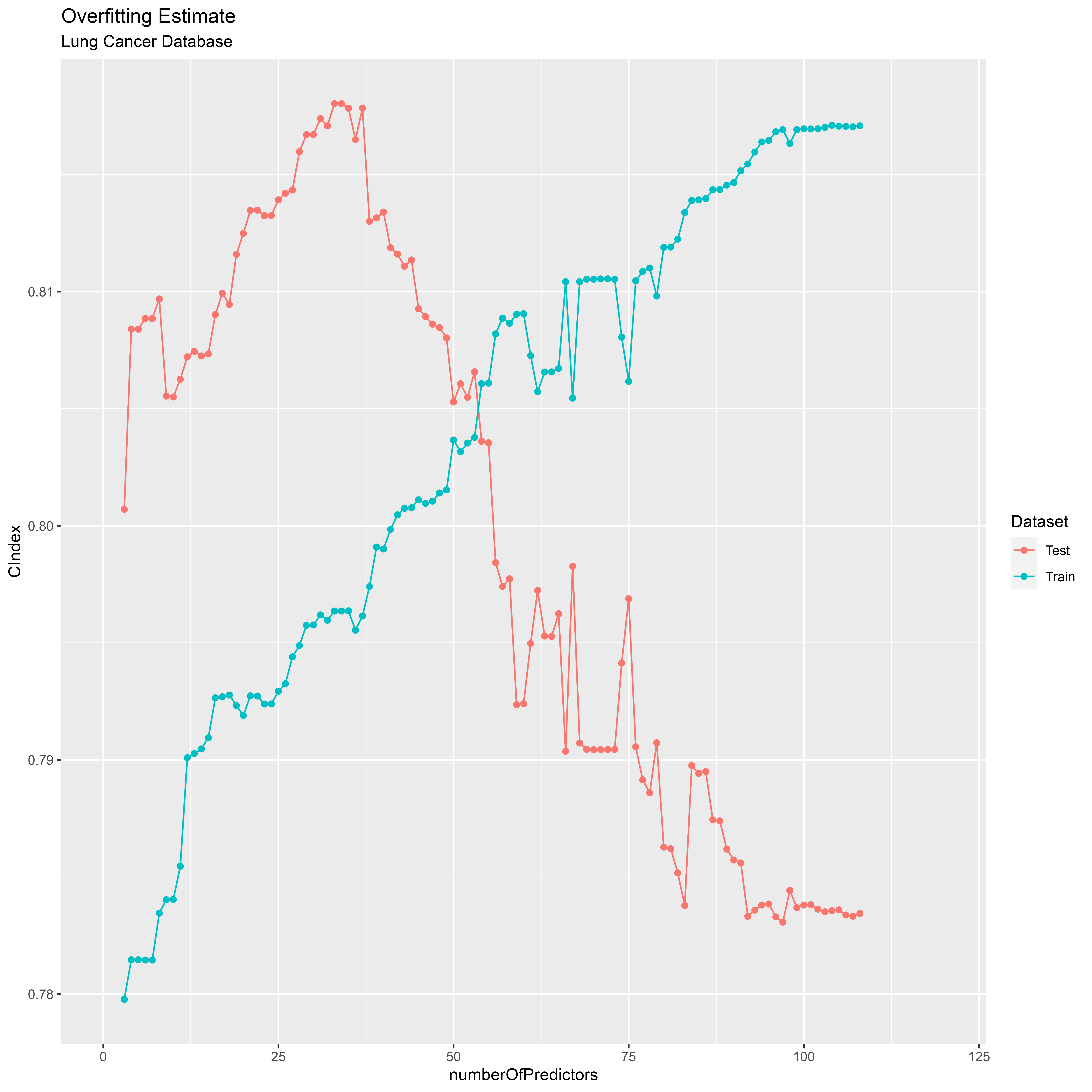

Supplement: Supplementary file 1 [file jcm-13-02613-s001.zip › Figure S3.png]
